# Small phytoplankton dominate western North Atlantic biomass

**DOI:** 10.1038/s41396-020-0636-0

**Published:** 2020-03-30

**Authors:** Luis M. Bolaños, Lee Karp-Boss, Chang Jae Choi, Alexandra Z. Worden, Jason R. Graff, Nils Haëntjens, Alison P. Chase, Alice Della Penna, Peter Gaube, Françoise Morison, Susanne Menden-Deuer, Toby K. Westberry, Robert T. O’Malley, Emmanuel Boss, Michael J. Behrenfeld, Stephen J. Giovannoni

**Affiliations:** 1 Department of Microbiology, Oregon State University, Corvallis, OR USA; 2 School of Marine Sciences, University of Maine, Orono, ME USA; 3 Monterey Bay Aquarium Research Institute, Monterey, CA USA; 4 Ocean EcoSystems Biology Unit, GEOMAR Helmholtz Centre for Ocean Research, Kiel, Germany; 5 Department of Botany & Plant Pathology, Oregon State University, Corvallis, OR USA; 6 Applied Physics Laboratory, University of Washington, Seattle, WA USA; 7 Laboratoire des Sciences de l’Environnement Marin, Institut Universitaire Européen de la Mer, Plouzané, France; 8 Graduate School of Oceanography, University of Rhode Island, Narragansett, RI USA

**Keywords:** Water microbiology, Microbial biooceanography, Biogeochemistry, Molecular ecology

## Abstract

The North Atlantic phytoplankton spring bloom is the pinnacle in an annual cycle that is driven by physical, chemical, and biological seasonality. Despite its important contributions to the global carbon cycle, transitions in plankton community composition between the winter and spring have been scarcely examined in the North Atlantic. Phytoplankton composition in early winter was compared with latitudinal transects that captured the subsequent spring bloom climax. Amplicon sequence variants (ASVs), imaging flow cytometry, and flow-cytometry provided a synoptic view of phytoplankton diversity. Phytoplankton communities were not uniform across the sites studied, but rather mapped with apparent fidelity onto subpolar- and subtropical-influenced water masses of the North Atlantic. At most stations, cells < 20-µm diameter were the main contributors to phytoplankton biomass. Winter phytoplankton communities were dominated by cyanobacteria and pico-phytoeukaryotes. These transitioned to more diverse and dynamic spring communities in which pico- and nano-phytoeukaryotes, including many prasinophyte algae, dominated. Diatoms, which are often assumed to be the dominant phytoplankton in blooms, were contributors but not the major component of biomass. We show that diverse, small phytoplankton taxa are unexpectedly common in the western North Atlantic and that regional influences play a large role in modulating community transitions during the seasonal progression of blooms.

## Introduction

Spring phytoplankton blooms in high-latitude oceanic regions are among the most-prominent natural events in the global ocean and have a profound impact on geochemical cycles [1, 2]. The annual phytoplankton spring bloom in the North Atlantic extends from 35° North to the Arctic Ocean [3], with the bloom peak progressing from February and March in the south to as late as July in the north [4].

Historically, diatoms have been recognized as the dominant taxa during the highest productivity stage of the North Atlantic bloom at high latitudes [5, 6]. Algorithms that predict carbon export from satellite-sensed chlorophyll often assign high export rates to phytoplankton blooms, on the assumption that large diatoms dominate at their climax [7]. A succession of coccolithophores, dinoflagellates, and pico-phytoplankton typically is expected to follow the diatom peak [8, 9]. This understanding of phytoplankton dynamics derives heavily from observations in eastern North Atlantic [10–13], but is extrapolated to the west, as if the North Atlantic region were a homogenous entity. However, the North Atlantic is heterogeneous in both space and time. For example, polar and tropical regions interact hydrographically while harboring distinctive phytoplankton communities [14] and a large westward gradient in eddy kinetic energy drives longitudinal heterogeneity by stirring and distorting the planktonic environment [15]. This creates a complex ecological landscape of dispersal and biological interactions [16] which is considered highly climate sensitive [17].

Comparatively few studies have investigated phytoplankton community composition in the North Atlantic during the winter transition, when the annual bloom is expected to initiate under the “disturbance and recovery” hypothesis [18, 19]. This early-winter initiation is triggered by physical mixing processes that dilute plankton populations and result in predator-prey decoupling. When mixed layer deepening ends, the bloom continues because improving growth conditions cause phytoplankton division rates to accelerate faster than rates of loss to predators [18–20]. The underlying ecological interactions between predators and prey, as stated in the “disturbance and recovery” hypotheses, underpin the spatio-temporal dynamics of communities across the seasonal trajectory of blooms, with global implications.

As part of the interdisciplinary North Atlantic Aerosols and Marine Ecosystems Study (NAAMES), we aimed to analyze patterns in western North Atlantic phytoplankton communities across seasons and latitude. We conducted two meridional transects from the subpolar to subtropical North Atlantic, one performed in early winter and one in spring [21]. NAAMES co-deployed multiple technologies for measuring phytoplankton, allowing us to assemble a synoptic view that resolved the full range of phytoplankton diversity at fine taxonomic scales. 16S rRNA V1–V2 amplicons were retrieved from five depths spanning the euphotic zone (5–100 m) in November 2015 and May 2016, at seven and five stations, respectively. Phytoplankton (plastids and cyanobacteria) 16S rRNA sequences were analyzed both by comparing amplicon sequence variants (ASVs) across stations and by phylogenetic methods based on a curated database. This approach to phytoplankton identification is timely given the climate sensitivity of this region and the increasing use of physiological and evolutionary aspects of cellular biology to understand bloom dynamics. Augmenting these data, cell counts and bio-volume concentrations from surface samples (5 m) were quantified using flow cytometry (FCM) and imaging flow cytometry (IFCB), respectively.

## Materials and methods

### Sampling

Two research cruises were conducted following a North to South meridional transect in the western north Atlantic Ocean onboard of the *R/V Atlantis*. Briefly, NAAMES 1 campaign took place in November 2015. Samples were taken from 7 stations along the jagged transect from 54 to 40° N and constrained to the 43–37° W region. NAAMES 2 took place in May 2016. Samples were taken from five stations along the jagged transect from 56 to 44° N and constrained to the 46–38 °W region. During NAAMES 2 campaign, station 4 was occupied for 4 days and sampled daily. A rosette water sampler equipped with 24 10-l Niskin bottles and a CTD (Sea-Bird 911+; standard conductivity, temperature and pressure sensors) was deployed down to 1000 m at dawn. In-situ nutrients and chlorophyll *a* were collected and processed as described in [22]. Near-surface (intake ~5 m) continuous temperature and salinity measurements were retrieved with a 1 min frequency using a thermosalinograph. Environmental data from the sampled stations used in this manuscript (photo1351_envdataV2.txt) are publicly available at github.com/lbolanos32/Phyto_NAAMES_2019.

### Mean dynamic topography

Mean dynamic topography (i.e., the 20-years average of sea surface height above geoid) was used to classify the observations collected during the NAAMES program. Maps of MDT were downloaded in the form of the MDT-CNES-CLS13 product that was produced by CLS and distributed by Aviso+, with support from CNES (https://www.aviso.altimetry.fr/). Different subregions were defined as in [23].

### Time progression of surface chlorophyll and mixed-layer depth

Surface chlorophyll data were based on MODIS-Aqua release R2018.0 processing. Eight-day averages of chlorophyll were plotted through the averaged meridional transect from January 2015 to January 2017. The MLD data are based on HYCOM’s global ocean salinity and temperature 3D models (using hindcast data). The density contrast used to define the mixed layer depth was 0.03 kg m^−3^. Both datasets were obtained from Oregon State University’s Ocean Productivity web site (http://sites.science.oregonstate.edu/ocean.productivity).

### Meridional displacement calculation and sea surface temperature

Altimetry-derived velocities (delayed time product) were downloaded from the Copernicus Marine Environment Monitoring Service (CMEMS, http://marine.copernicus.eu) and used to backtrack the origin of water parcels using the LAMTA Lagrangian scheme [24, 25]. The meridional displacement over 30 days was then calculated as *latitude(t)* *–* *latitude (t-30)*. The obtained map has a resolution of 1 km, but it is based on geostrophic velocities measured at ~ 25 km resolution. Consequently, identified patterns are representative of the large-scale geostrophic circulation, the mesoscale, and of some submesoscale stirring induced by mesoscale features. Displacement caused by intense wind events and vertical movements cannot be captured by this approach. More details can be found in [23]. Multi-scale Ultra-High Resolution Sea Surface Temperature (MUR-SST) data were downloaded from the MUR-JPL website https://mur.jpl.nasa.gov/index.php (US NASA Jet Propulsion Laboratory Physical Oceanography Distributed Active Archive Center (JPL PO.DAAC) (2011). GHRSST Level 4 MUR Global Foundation Sea Surface Temperature Analysis (v4.1) (GDS versions 1 and 2). National Oceanographic Data Center, NOAA. 10.5067/GHGMR-4FJ01. 2019-02-24). This specific product is distributed daily with a nominal resolution of 1 km and it combines observations from different infrared and microwave satellites and in-situ buoys.

### DNA extraction and amplicon sequencing

Four liters of water was collected from the rosette casts for eight different depths (5, 25, 50, 75, 100, 150, 200, and 300 m) in a polypropylene carboy (rinsed three times). Microbial biomass was collected on a 0.22-µm pore-size Sterivex filter (polyethersulfone membrane, Millipore, Burlington, MA, USA) using an eight-channel peristaltic pump (flow-rate 30 ml/min) (Table S1). One mililiter of sucrose lysis buffer was added to the filters and stored at −80°. Nucleic acids were extracted using a phenol:chloroform protocol described previously [26, 27].

Amplification of the V1–V2 region of the 16S rRNA gene was performed using the 27 F (5′-AGAGTTTGATCNTGGCTCAG-3′) and 338 RPL (5′-GCWGCCWCCCGTAGGWGT-3′) primers attached to their respective overhang adapters following the standard 16S sequencing library preparation protocol conditions (Illumina Inc.).

Libraries for each amplicon reaction product were done attaching dual indices and Illumina sequencing adapters with the Nextera XT Index Kit (Illumina Inc.) using a second PCR amplification (following manufacturer conditions). Purified libraries were pooled in equimolar concentrations for each campaign (56 samples for NAAMES 1 and 64 for NAAMES 2). Each pool was sequenced using the Illumina MiSeq platform (reagent kit v.2; 2×250 PE; Illumina Inc.) at the Center for Genome Research and Biocomputing (Oregon State University, Corvallis, OR, USA).

### 16S rRNA gene amplicon analyses

Primer sequences were cropped out using the CutAdapt software [28] removing a fixed number of bases (-u parameter) matching the 27 F (20 bp) and 338 RPL (18 bp) primer length. Trimmed fastq files were quality filtered, dereplicated and merged with dada2 R package, version 1.2 [29]. ASV table was constructed with the makeSequenceTable command and potential chimeras were removed de novo using the removeBimeraDenovo command. Taxonomic assignment of the ASVs was determined using a two-step approach. First, with the assignTaxonomy command in dada2 package and the Silva database (version 123) as reference. Second, plastid and cyanobacteria ASVs were extracted and phylogenetically placed in a curated cyanobacteria and plastid reference tree [30, 31] using Phyloassigner version 089 [32]. ASVs were aligned against the nonredundant nucleotide NCBI database using blastn [33], excluding environmental samples. Plastid ASVs with an identity of 99% or greater to a reference sequence were annotated down to genus. We considered only samples above 100 m which had ≥1600 plastid and cyanobacteria amplicons. ASVs hierarchical clustering was done with the function hclust using normalized data with the negative binomial Wald implemented in DESeq2 [34]. Observed composition, alpha-diversity indexes, and bray-curtis dissimilarities were calculated from subsampled datasets rarefied to 1594 sequences using Phyloseq version 1.2.0 [35]. Bray-curtis dissimilarities were used to generate a principal coordinate analysis. Taxonomy relative contribution bar and pie plots and total chlorophyll *a* heat map (Fig. 2) were done with ggplot2 package [36] and edited in inkscape (www.inkscape.org) for esthetics. Mixed layer depth calculations used in Fig. 2b are described in [37].

Intersecting sequence variants analysis was done with upsetR package [38] and the differential abundance analysis with DESEq2 (alpha cutoff < 0.01). Principal component analysis of upper water column (≤100 m) physical and chemical characteristics was conducted using the *prcomp* function of the R Stats package. A detailed pipeline and the generated files during the analysis are provided in https://github.com/lbolanos32/Phyto_NAAMES_2019. Short read sequence files are publicly available in the SeaWiFS Bio-optical Archive and Storage System (SeaBASS, https://seabass.gsfc.nasa.gov/investigator/Giovannoni,%20Stephen) as associated files.

### Flow cytometry cell counts

Four milliliters of unpreserved surface (5 m) seawater samples were collected from rosette casts into sterile 5 ml polypropylene tubes (rinsed three times) and immediately stored at ∼4 °C in the dark until analysis (Table S1). BD Influx Cell Sorter (ICS) (Becton Dickinson Biosciences, Franklin Lakes, NJ, USA) was used to enumerate and classify phytoplankton groups [39–41]. Cells were identified based on fluorescence emissions at 692 and 530 nm and forward and side scattering intensity. Sample tubes were kept shaded, but not completely dark during analysis using opaque tape [42, 43]. A minimum of 7000 total cells were interrogated per sample. Flow rates were calculated from volumetric changes in a 1 ml water sample over a known time (60 s or greater) using a pipettor to determine the volume of water lost. This was performed immediately following the analysis of samples collected at each time point. The ICS was calibrated daily with fluorescent beads (Spherotech, SPHEROTM 3.0 μm Ultra Rainbow Calibration Particles, Becton Dickinson Biosciences) following the manufacturer’s standard protocols. Flow cytometry data were organized into four major phytoplankton groups: *Prochlorococcus*, *Synechococcus*, pico-eukaryotes, and nano-eukaryotes based on the grouping of cells with regard to intensity of fluorescence and forward scattering properties.

### Imaging FlowCytobot data retrieval and analysis

Digital images of nano- and micro-phytoplankton from surface samples were obtained at each station using an Imaging FlowCytobot (McLane Labs, Falmouth, MA, USA). The optical and fluidic design of the IFCB has been described in [44]. The intake tubing of the IFCB was inserted into the main flow-through system of the boat and 5 mL samples were automatically drawn every ~25 min. A 150 μm Nitex mesh was placed on the intake to prevent the entrance of large particles that could clog the flow cell of the IFCB. The camera was triggered by the autofluorescence of cells and the lower size limit for detection was set by fluorescence threshold to trigger the camera. A comparison of cell abundances in the nano-size range between FCM and IFCB indicated that the IFCB likely underestimated cells < 8 μm (data not shown), and therefore only cells >8 μm are included in the IFCB data analysis (Table S1). Images were processed using custom software as described in [45, 46]; codes are available at https://github.com/hsosik/ifcb-analysis/wiki. Processed images, metadata, and their associated features (equivalent spherical diameter, area, volume and other morphometric parameters derived during image processing) were uploaded to EcoTaxa ecotaxa.obs-vlfr.fr [47]. Classification of the image collection into taxonomic and other functional groups was done using a subset of manually annotated and classified images. This subset was classified into living (~100 different groups) and nonliving particles. Living particles have a taxonomic resolution ranging from genus to class. This manually annotated data were the training set for automatic prediction of the remaining images affiliation (a total of 380,382 images), using a random forest algorithm. Computer predictions were validated manually and particles were re-assigned to the proper taxonomic group when needed. For community composition analysis, several water samples from each station were pulled together to ensure sufficient number of cells per samples (sample size of at least 3000 cells).

## Results

### Mean dynamic topography (MDT) delineates phytoplankton regional variation

**Fig. 1:**
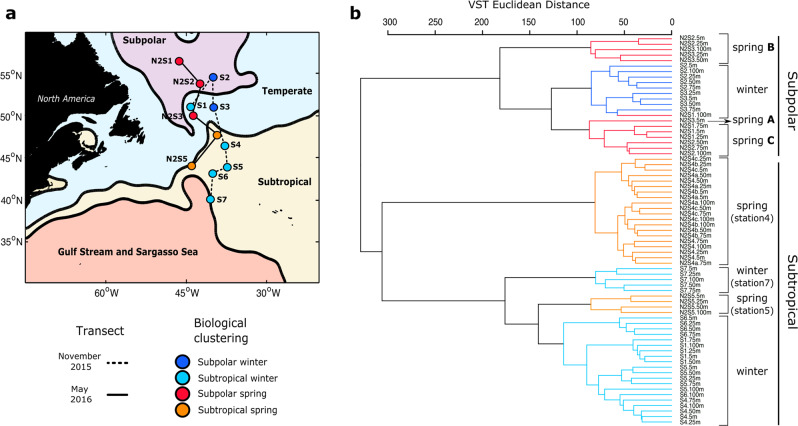
Map of the sampled stations in the North Atlantic and hierarchical clustering of the samples based on the ASV profiles. **a** Map of the western North Atlantic showing the subregions established by the mean dynamic topography analysis. Stations are indicated as circles. Those joined by a solid line were sampled in November 2015 (winter) and by a dashed line in the following spring (May 2016). Stations are color coded by the categorical sample assignments determined in the ASVs hierarchical clustering analysis as shown in (**b**): Subpolar winter, Subpolar spring, Subtropical winter, and Subtropical spring. **b** ASVs dendogram defined by hierarchical clustering of samples collected from the upper 100 m water column in winter and spring. Branches of the dendogram colored in navy blue and light blue represent samples collected in early winter from the Subpolar and Subtropical regions of the study area, respectively. Branches colored in red and cyan represent samples collected in the spring from the Subpolar and Subtropical regions, respectively. Within the subpolar category, three spring groups were defined. Spring ‘A’ represents the surface of the most southern subpolar station. Spring ‘B’ represents samples below the MLD at station 3 and above at station 2. Spring ‘C’ represents samples below the MLD at station 2 and above at station 1 (expanded in Fig. 2b).

MDT divides the North Atlantic into four regions: *subpolar*, *temperate*, *subtropical*, and *Gulf Stream/Sargasso Sea* (Fig. 1a) (ref. [23]). Two meridional transects covered these four subregions capturing the initiation of winter deep mixing and the peak of the bloom following the water column re-stratification (Fig. S1).

Genetic profiles were retrieved from the upper 100 m at each station. Normalized ASV counts were used to establish a comparable unit of measurement and calculate a standardized Euclidean distance matrix. Distances representing the similarity between samples were grouped using a hierarchical clustering approach. In hierarchical clustering of phytoplankton ASV frequencies, samples from the same region grouped more closely than samples from the same season (Fig. 1b), indicating that properties associated with water masses strongly influence community composition. Hierarchical clustering did not discriminate *subpolar* and *temperate* regions (Fig. 1b), so hereafter profiles from these stations are considered as a single *subpolar* region. Likewise, *Gulf Stream/Sargasso Sea* clustered with and was combined with the *subtropical* region and generally exhibited an ASV profile distinct from the *subpolar*. The major division observed was between the *subpolar* and *subtropical*. Temperature and salinity (T–S) in near-surface water varied between MDT subregions, but were indistinguishable within the subpolar and subtropical (Fig. S2). An anomalous phytoplankton community that did not fit this pattern, winter station 1 (43° W, 51° N), was physically located in the temperate subregion, but its ASV profile clustered with the *subtropical* stations. Satellite altimetry and sea surface temperature showed that this station was in an anticyclonic eddy that originated in the subtropics (Fig. S3). T–S confirmed that winter station 1 conditions were similar to those of the subtropics (Fig. S2). These findings illustrate the dynamism of North Atlantic hydrography and the importance of transport as a factor contributing to phytoplankton community structure.

Statistical ordination of physico-chemical water properties clustered the stations similarly to the community-based ASV hierarchical clustering, a further indication that phytoplankton community structure is shaped by habitat variables (Figs. S4, S5, S6, S7). In winter, salinity, temperature and the ratio of silicate to total-dissolved-nitrogen (Si:DIN) generally increased from north to south, with the exception of station 1 of subtropical origin, whose physico-chemical properties aligned with station 4 (Fig. S4). In spring, discrete high nutrient availability, low temperature, and salinity distinguished *subpolar* stations (Fig. S4). As expected, these nutrient differences were reflected not only in shifts in ASV-based community composition, but also in overall surface chlorophyll concentrations (Fig. S5). These shifted from low chlorophyll (0.2–0.5 mg/m^3^) in winter, except station 3 (1.1 mg/m^3^), to higher levels in spring (0.4–3.5 mg/m^3^). Values at subtropical stations were lower and shifted less over the seasonal transition, ranging from 0.4 to 1.7 mg/m^3^. The maximum values observed were in spring in the subpolar region (2.7–3.5 mg/m^3^).

The above findings suggest that, in the western North Atlantic, the differences in abiotic factors that delimit regions also create dynamic ecological borders for phytoplankton. This physico-chemical structuring of communities is referred to as “environmental filtering” [48] and it implies that the distribution of specific communities can be predicted from an extensive description of the environment. Alternate perspectives, considered further below, place more weight on biological factors in shaping communities.

### Seasonal water column dynamics shape phytoplankton communities within the regional variation

**Fig. 2:**
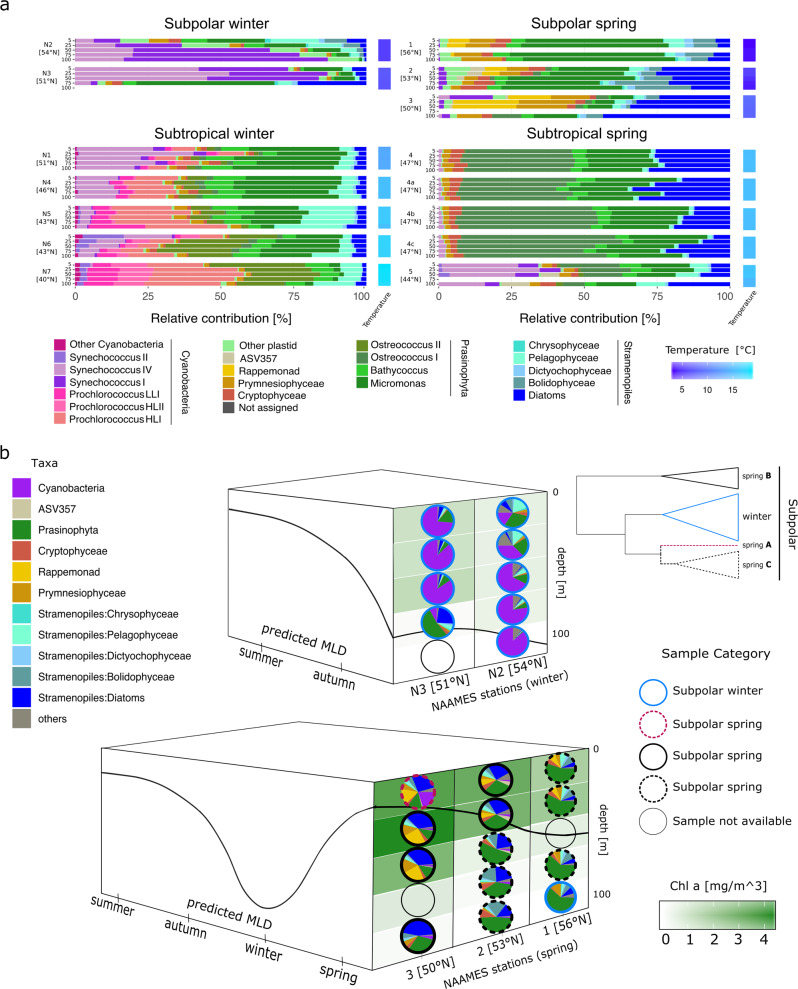
Taxonomic and ecological description of the 16S rRNA phytoplankton amplicon datasets. **a** Relative contributions of phytoplankton taxa for depth profiles at each station. Water column is represented by bars indicating five sampling depths (5, 25, 50, 75 and 100 m) and arranged from surface to deep samples. Stations are organized by the categories defined in the ASVs hierarchical clustering (Fig. 1b). Station 4 in the spring was occupied 4 days, capturing a rapid water column re-stratification event. These profiles are labeled as 4 (May 24), 4a (May 25), 4b (May 26) and 4c (May 27 2016). Water column temperature gradient is depicted as a heat map on the right side of each station bar plots. (**b**) Diagram depicting the spatio-temporal shifts in the subpolar region phytoplankton community composition, derived from phylogenetic taxonomic assignments. Top: vertical structure of community composition in November 2015, bottom: vertical structure of community composition in May 2016. Height of each box represents depth (0–100 m) and the solid black line represents the MLD. Left side of each box depicts the predicted annual dynamics of the mixed layer. Right side represents MLD (black line) and latitude, most southern in the front and most northern in the back. Vertical distributions of chlorophyll *a* concentration are represented by the background shades of green. Circle periphery of the pies identifies each sample to any of the defined subgroups from the ASVs hierarchical clustering analysis: winter, spring ‘A’, spring ‘B’ and spring ‘C’. A simplified representation of the ASVs clustering dendogram (subpolar section, Fig. 1b) is shown on the top-right corner.

To analyze the seasonal effect within the defined *subpolar* and *subtropical* regions, we compared phytoplankton community composition between winter and spring samples using phylogenetic methods that assign ASVs to taxonomic categories [30, 31]. *Cyanobacteria* and pico-phytoeukaryotes numerically dominated the ASVs in the western North Atlantic during winter (Fig. 2a). In the subpolar region, *Cyanobacteria* in *Synechococcus* clades I and IV represented >50% of the ASVs, while the eukaryotic pico-prasinophyte genera *Bathycoccus* and *Micromonas* were also notable (>10%).

In the subtropical winter samples, most *Cyanobacteria* were in the clades *Synechococcus* IV, *Prochlorococcus* high light I and II, and low light I. *Prochlorococcus* relative amplicon contributions increased with decreasing latitude alongside decreases in *Synechococcus* clades IV and II at the southernmost subtropical stations (stations 5–7). Among eukaryotic phytoplankton, *Bathycoccus* and *Micromonas* were again notable (being >30%), with smaller contributions from stramenopile, cryptophyte, and prymnesiophyte algae. Surprisingly, the recently recognized pico-prasinophyte species *Ostreococcus* Clade OII was also prominent [49], especially moving southwards. A similar apparent jump in OII contribution has been observed at the border of the Kuroshio Current and the Subtropical North Pacific Gyre [50]. An additional surprise was that stramenopiles formed a relatively small part of the phytoplankton community at both *subpolar* and *subtropical* winter stations. Moreover, among stramenopiles, the relative amplicon contribution of pelagophytes was higher than that of diatoms, although the latter are typically considered important in high latitude bloom scenarios. Taken together, these observations indicate that pico-size phytoplankton dominated winter conditions. Distinctive communities matched the defined subregions and showed smooth latitudinal taxonomic transitions within them. However, strong dynamic mesoscale features such as those found at station 1 can disrupt these ecological boundaries.

Spring in both regions was characterized by a major reduction of *Cyanobacteria* and a shift to eukaryote-dominated communities with differences in taxonomic composition from the winter period (Fig. 2a). At subpolar spring stations, the relative contribution of pico-phytoeukaryotes, largely *Micromonas*, a genus which has been shown to be increasing in the Arctic in association with climate-change [51], decreased from 60% of amplicons to <15%, in a north-to-south trend. Relative contribution of diatoms, prymnesiophytes, rappemonads, [52] and cryptophytes, increased from north to south. We observed considerable variability between the communities at the two subtropical spring stations. Station 4 was consistently dominated by pico-phytoeukaryotes, of which *Ostreococcus* Clade OI had the highest relative contribution, but other pico-prasinophytes were also numerous. Eukaryotic phytoplankton were similar at stations 4 and 5, but there was a higher relative contribution of *Synechococcus* (>30%) at station 4, largely clade IV. Thus, in the transition from winter to spring in the western North Atlantic, communities shifted unexpectedly from being dominated by pico-phytoplankton to a diverse assemblage of eukaryotic phytoplankton.

NAAMES cruise tracks were latitudinally oriented and thus captured the seasonal progression of blooms in a time span of days, which otherwise would take weeks for a ship stationed at constant latitude (Fig. 2b). Ordination of community composition revealed dramatic shifts between winter and spring across subregions (Fig. S8). Because of a homogeneously mixed water column, winter ASV depth profiles clustered tightly by station, and followed a latitudinal gradient of dissimilarity. Among spring samples, latitudinal shifts in phytoplankton community structure were evident, but the gradient was uneven. Specifically, subpolar phytoplankton communities were not strictly clustered by station. Instead, they followed a pattern in which communities below the MLD clustered preferentially with those above the MLD of the nearest northern station, suggesting that progressing stratification and associated environmental parameters overtake the influence of surface parameters, as observed in other marine environments [53, 54].

### Some phytoplankton benefit from disturbance and recovery dynamics

Within the spatio-temporal phytoplankton community variation, we investigated how ASVs were distributed across regions and seasons (Fig. S9). We identified ASVs enriched in spring and classified them as *winter-detected* if they were present in both seasons, or *winter*-*undetected* if they were absent in winter datasets (Fig. S10 and Table S2). Winter-detected ASVs may represent taxa that benefit from the disturbance and recovery effect [19], growing in winter and contributing to the bloom climax. In the subpolar region, 52 ASVs (21.9% of the total in the region) were significantly abundant in spring (*p* < 0.01) relative to winter. Of these, the 27 (52%) winter-detected variants were composed mostly of diatoms and prasinophytes. (Fig. S10). Interestingly, three of the most represented ASVs in subpolar spring, *Chaetoceros* (ASV134) and the rappemonad variants (ASV193 and ASV195), did not have a winter representative and were categorized as winter-undetected. In the subtropical region, 57 ASVs (15.7% of the total in the region) were significantly abundant in spring (*p* < 0.01). Of these, 27 (47.3%) were detected in winter; these were composed mostly of diatoms, prasinophytes, and chryptophytes. Among the diverse winter-detected organisms common in both regions were *Pheocystis*, *Teleaulax*, *Minutocellus*, *Thalassiosira*, *Micromonas* E2, and *Ostreococcus* Clade OI. Nondetection in the winter of approximately half of the ASVs that were significantly enriched in spring suggests either high differential success for these taxa in the winter-to-spring transition, or transport with water masses.

### Pico- and nano-phytoeukaryotes contribute the most to biomass

**Fig. 3:**
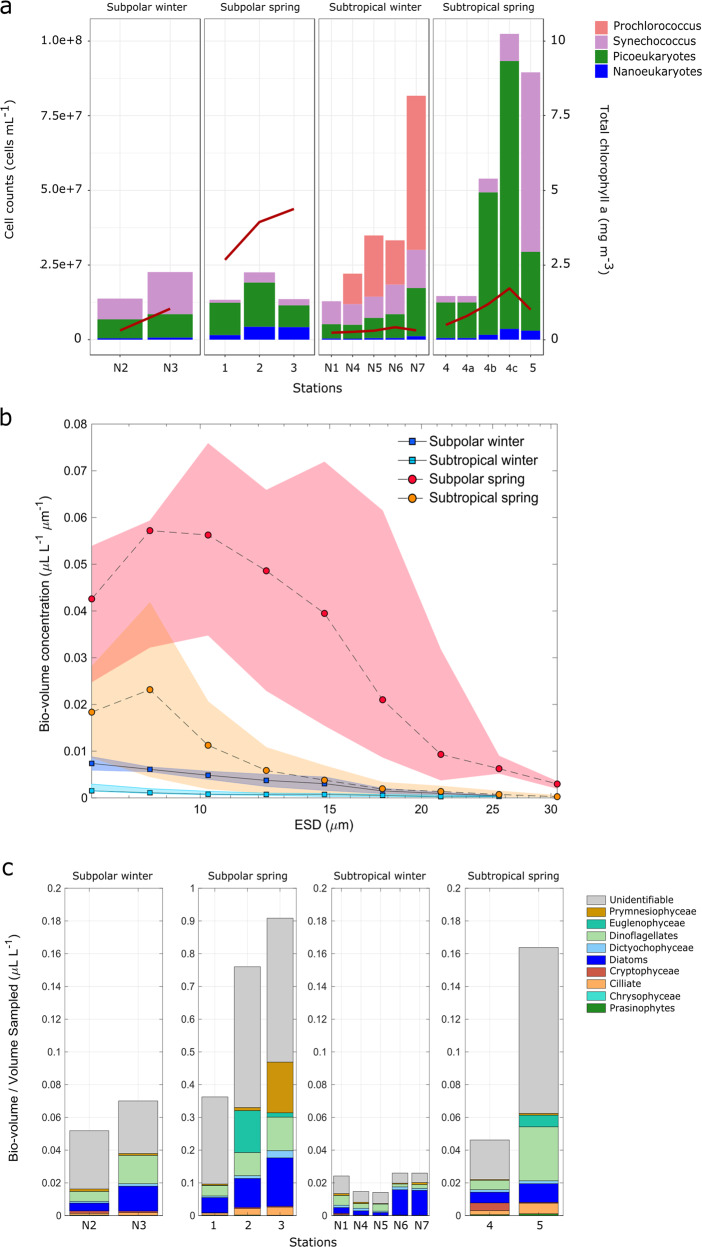
Cell-size and bio-volume characterization of the phytoplankton community using flow cytometry and IFCB. **a** Flow cytometry cell counts of surface samples. Each bar plot represents the stacked number of cells (right *y*-axis) for the different taxonomic categories analyzed. Bar plots are organized in four panels grouped by the defined categories. Total chlorophyll *a* concentrations are shown as an overlapped red line (left *y*-axis). (**b**) Total bio-volume distributions of chlorophyll containing taxa collected at the surface (ship intake) and derived from the IFCB images (fraction of cells >8-µm diameter) at each station for both campaigns. Data points and lines indicate the bio-volume average for each category. **c** Total cell bio-volumes derived from IFCB images (fraction of cells >8 µm diameter). Colors represent the volume contributions of major taxonomic groups. Cells that could not be identified are grouped under the ‘unidentifiable’ category. It should be noted that size fractions differ between the data sources depicted. The FCM data typically represent only cells <40 µm, IFCB captures taxa between 8 and 100 µm, while total chlorophyll *a* measurements were performed on whole water samples and therefore represent whole community biomass.

Perhaps the most surprising observation from the NAAMES campaigns is the unexpectedly low abundance of large phytoplankton cells (micro-phytoplankton; >20 µm) in the spring, including stations where phytoplankton biomass was high. Multiple lines of evidence support this observation. In addition to the numerical predominance of known pico- and nano-phytoeukaryotic taxa in genetic profiling analyses, FCM and IFCB show that the dominance is not only in abundance but also in terms of contribution to total bio-volume (the contribution of phytoplankton to biomass as a function of cell size) (Fig. 3). FCM cell counts in surface samples confirmed high abundances of *Cyanobacteria* and pico-phytoeukaryotes in the subpolar winter (Fig. 3a). Moreover, the FCM data clearly aligned with *Prochlorococcus* and *Synechococcus* ASV frequencies across the regions in both seasons. At subtropical spring station 4, FCM revealed that pico- and nano-phytoeukaryotes were the dominant blooming populations responding to rapid water column re-stratification over the 4-day occupation of this station [41].

Across the NAAMES transect, spring high biomass communities were composed mainly of cells in the pico and nano-size phytoplankton (Figs. 3b, S11 and S12). Bio-volume distributions in surface samples (ship-intake depth) were constructed from curated IFCB data (Figs. 3b and S12). Individual bio-volume distributions by size (Fig. S12) suggested that elevated abundances of nano and lower-end micro size cells (ca. 10–30 µm) likely explain the higher chlorophyll values observed at subpolar stations, relative to pico-phytoplankton cell counts measured by flow cytometry. At the two northern stations, cells of ca. 10-µm diameter were the dominant size class contributing to bio-volume per liter. This trend shifted at the most southern subpolar station 3 to slightly larger cells, where the bio-volume dominant fraction was around 15-µm diameter. At subtropical stations (4 and 5), bio-volumes followed a unimodal distribution, peaking near the 8-μm lower quantitative threshold for IFCB. Averaged bio-volumes by subregion and season (Fig. 3b) showed increased phytoplankton bio-volumes in spring in both regions, as expected. The magnitude of the increase was drastically higher in the subpolar region. Bio-volumes in winter and the subtropical spring were below 0.17 µL/L, while the subpolar spring stations showed a north-to-south increase ranging from ~0.37 µL/L (station 1) to 0.9 µL/L (station 3).

The taxonomic contributions of nano and micro (>8 µm) phytoplankton to the measured bio-volumes were derived from the morphological characterization of IFCB high-throughput image data (Fig. 3c). Within this size-fraction, diatoms and dinoflagellates composed the major bio-volume fraction through regions and seasons. In the high biomass subpolar spring, diatoms and dinoflagellates increased with decreasing latitude. In addition to this trend, some taxa emerged as high contributors at specific stations, such as euglenophytes at station 2 and prymnesiophytes at station 3. ASVs for the major diatom taxa present, *Chaetoceros* and *Minutocellus*, the prymnesiophyte *Phaeocystis*, and other two variants (ASV193 and ASV195) annotated as rappemonads, which are thought to be in the 3–10 µm size range, followed a similar trend of increasing from north to south in the subpolar spring. The co-occurrence of well-known blooming *Chaetoceros* and colony forming *Phaeocystis* (detected by ASVs and IFCB), along with nano-size taxa, such as *Minutocellus* (detected by ASVs), and pico-phytoeukaryotes (detected by FCM and ASVs), in the highest chlorophyll samples of the subpolar spring, suggests that assumptions of large diatom dominance during the bloom in the western North Atlantic may arise either from the use of methods that do not capture or identify smaller eukaryotic phytoplankton cell types or from historical sampling biases favoring the eastern North Atlantic.

## Discussion

A synoptic view of phytoplankton diversity emerged from the multiple technologies co-deployed on NAAMES. Not surprisingly, the North Atlantic held richly complex assemblages of phytoplankton, but unanticipated was the regional complexity of phytoplankton communities and their predictability from season and MDT. Compelling evidence for seasonality in photosynthetic plankton community structure previously has emerged from time-series studies [55–57].

NAAMES findings support that phytoplankton community variation across geographical regions as complex as the North Atlantic can partially be predicted in a context of complexly resolved hydrographical schemes [58]. Furthermore, latitudinal sampling enabled us to reconstruct the seasonal progression of the bloom. Our results suggest that spring community shifts are strongly associated with water column stratification and physico-chemical changes accompanying the stratification process. We found that the seasonal progression of changing daylength and the regional origins of the water masses were powerful modulators of community composition. However, this finding does not necessarily imply that the physico-chemical influences drive community composition through simple ‘bottom up’ mechanisms. It is more likely that interactions exist between physico-chemical factors and biotic factors, where ‘bottom up’ modulation of phytoplankton community structure is paralleled by modulations in zooplankton predation [59], viral infections [60], interactions among the microorganisms [61], and neutral turnover among ecologically similar taxa [62].

Diatoms, which are often thought to dominate phytoplankton blooms [8, 63], were infrequently a major fraction of the phytoplankton genetic profiles and, when diatoms were a relatively high fraction of the ASVs (subpolar spring stations 2 and 3), IFCB data showed that they were mostly small diatoms in the nano-phytoplankton or at the lower end of the micro-phytoplankton size category. Biogeochemical models are often influenced by the perception that North Atlantic phytoplankton blooms are composed of large cells that contribute massively to export carbon flux [64]. This perception has been perpetuated by models that assume that diatoms are uniformly large cells. However, diatoms are diverse in size, introducing substantial variation in their contribution to the export of carbon [63, 65]. Support for our findings can be found in previous reports that noted small phytoplankton cells are common components of the North Atlantic spring bloom [1, 66].

The sharp decline of cyanobacteria populations in the spring indicated that they were potentially outcompeted by eukaryotic taxa as daylength increased. Indeed, deep mixing and associated physico-chemical parameters appeared to serve as a springboard for pico- and nano-phytoeukaryotes, which prospered in the late spring. Pico- and nano-phytoeukaryotes stood out in their persistence as large populations across regional geography and the dynamic variation associated with local blooms and mesoscale features.

Statistical analysis of significantly abundant ASVs composing the spring bloom revealed that around half of these sequences could not be genetically traced to the winter samples. This suggests that there are life history strategies by which phytoplankton that are undetectable in winter can rise to high numbers in the spring or there is a quick community turnover due to water masses circulation. Although transport influences community composition on regional scales, as we observed at winter station 1 or as observed at a global scale in the Agulhas rings system [67], the effects on these circulation systems in a warming future has been largely overlooked.

Whether NAAMES observations of small phytoplankton in the western North Atlantic, including multiple taxa never before documented in this environment, are due to physical differences between the western and eastern North Atlantic [68], ocean warming and higher atmospheric CO_2_ concentrations [69, 70], constraints of previously applied methodologies, or are a coincidental annual anomaly, is still to be determined. Of these explanations, which are not mutually exclusive, differences between the west and east Atlantic in eddy kinetic energy, mixed layer depth, photosynthetic active radiation and the influence of arctic water masses, have been proven by previous studies to affect composition and biomass of the phytoplankton blooms [67, 71]. If our results are representative of the broader western North Atlantic, then they have major implications on current understanding of phytoplankton bloom impacts on regional carbon biogeochemistry. Specifically, multispecies blooms, such as those described here, can have lower carbon export efficiencies than mono-specific blooms of diatoms or prymnesiophytes [72]. Populations dominated by smaller phytoplankton may also be associated with marine food webs that have lower trophic efficiencies than those based on larger phytoplankton [73, 74]. Although not explored in this study, ecological interactions between heterotrophic microorganisms, mixotrophic protists, and autotrophic primary producers likely play important roles in the development and progression of seasonal phytoplankton blooms. While further studies are needed to evaluate the full implications of NAAMES data, results presented here contrast with expectations based on previous reports, revealing both structured and dynamic aspects of the system. Notably, the profoundly contrasting composition of the winter community, and the diverse phytoplankton assemblages dominated by small taxa we found in the spring, are system features that alter our perspective and will likely set the stage for future research.

## Supplementary information

Supplementary material
