# Supplementary material for: Small phytoplankton dominate western North Atlantic biomass
Source: ISME J. 2020 Mar 30;14(7):1663–74. doi: 10.1038/s41396-020-0636-0 (PMC7305139; doi:10.1038/s41396-020-0636-0)
Supplement: Supplementary file 1 — Supplementary material [file 41396_2020_636_MOESM1_ESM.pdf]

Small phytoplankton dominate western North Atlantic biomass
Supplementary Material

Luis M. Bolaños[1], Lee Karp-Boss[2], Chang Jae Choi[3,4], Alexandra Z. Worden[3,4], Jason R. Graff[5], Nils Haëntjens[2], Alison P. Chase[2], Alice Della Penna[6,7], Peter Gaube[7], Françoise Morison[8], Susanne Menden-Deuer[8], Toby K. Westberry[5], Robert T. O'Malley[5], Emmanuel Boss[2], Michael J. Behrenfeld[5], Stephen J. Giovannoni[1]*

[1] Oregon State University, Department of Microbiology, Corvallis, OR, USA [2] University of Maine, School of Marine Sciences, Orono, ME, USA [3] Monterey Bay Aquarium Research Institute, Monterey, CA, USA [4] GEOMAR Helmholtz Centre for Ocean Research Kiel, Ocean EcoSystems Biology Unit, Kiel, DE [5] Oregon State University, Department of Botany & Plant Pathology, Corvallis, OR, USA [6] Applied Physics Laboratory, University of Washington, Seattle, WA, USA [7] Laboratoire des Sciences de l'Environnement Marin (LEMAR), UMR 6539 CNRS-Ifremer-IRD-UBO-Institut Universitaire Européen de la Mer (IUEM) Plouzané, FR [8] University of Rhode Island, Graduate School of Oceanography, Narragansett, RI, USA

*Corresponding author
E-mail: Steve.giovannoni@oregonstate.edu

This file includes:
Supplementary Tables S1-S2
Supplementary Figures S1-S12

TABLE I:
Table S1 Summary of the methods used to characterize the community composition.

Method	Target	Units	Size thresholds	Analyzed samples
Genetic profiling (16S rRNA amplicon sequencing)	Photosynthetic origin genes (Cyanobacteria and plastids; dinoflagellates systematically underrepresented)	Relative abundance (%)	NA (biomass filtered with a 0.22 μm pore size)	Stations (5,25,50,75,100 m depths)
Flow Cytometry	Cells	Cells per milliliter (cells mL^{-1})	>40 μm	Stations (5m)
Imaging Flow Cytobot (IFCB)	Phytoplankton cells	Size distribution expressed in bio-volume (μL L^{-1})	> 8 μm and <100 μm	Stations (5m)
Imaging Flow Cytobot (IFCB)	Phytoplankton cells	Taxonomic distribution expressed in bio-volume (μL L^{-1})	>8 μm and <100 μm	Stations (5m)

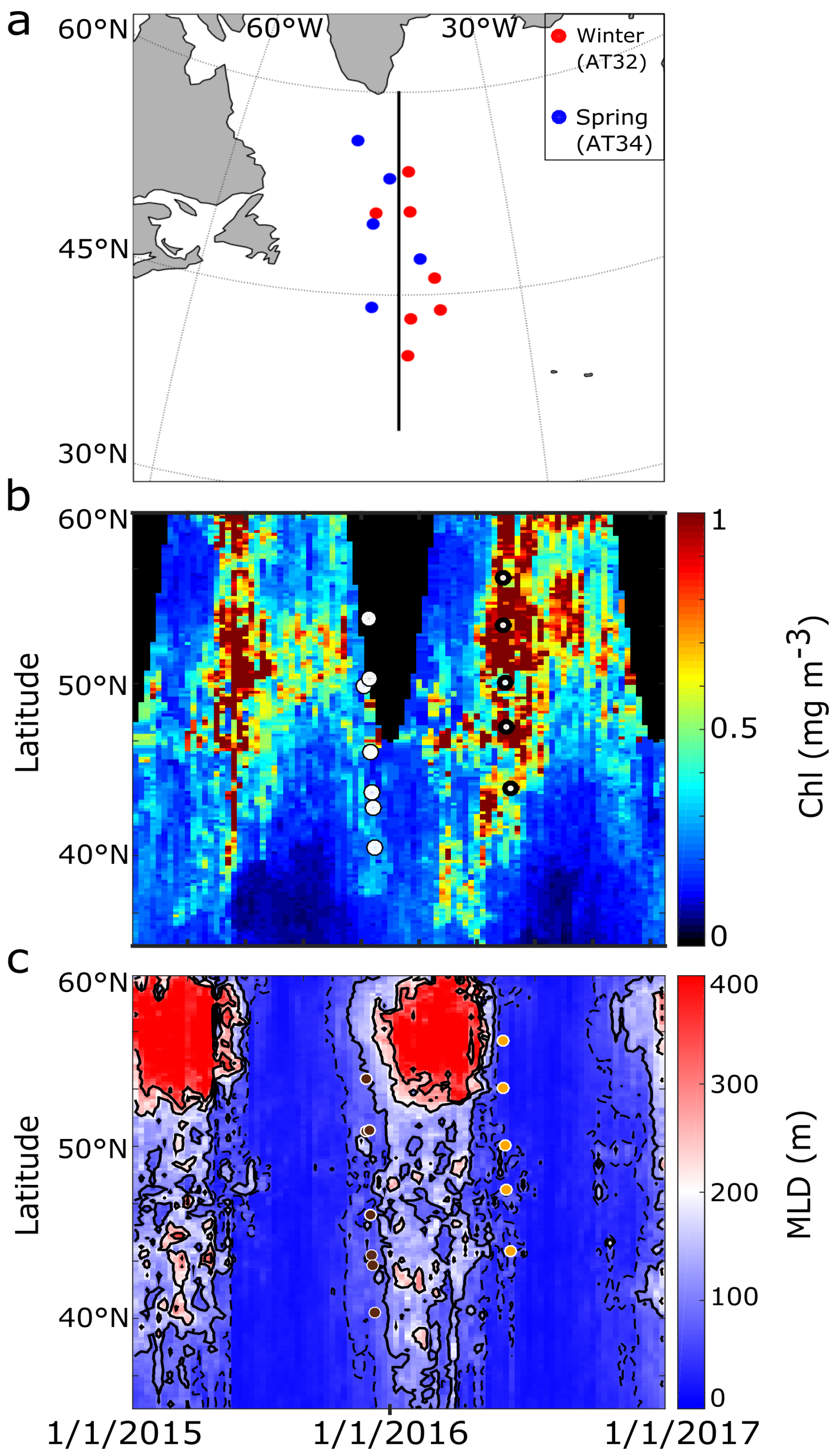

Figure S1 Surface chlorophyll and mixed-layer depth time progression of the averaged transect for the NAAMES campaigns. (a) Map of the stations sampled through winter (November 2015) and spring (May 2016) NAAMES campaigns. Winter and spring stations are depicted as red and blue circles, respectively. The locations of the stations were used to calculate an average longitudinal transect represented by the black line. (b) Filled surface chlorophyll satellite data time progression in the averaged transect from January 2015 to January 2017. Eight-day averages of chlorophyll are shown in the x-axis and through the latitude of the meridional averaged transect represented in the y-axis. Heatmap scale is shown from 0 to 1 mg/m^3. White and black-bold circles represent the winter and spring stations, respectively. (c) Mixed-layer depth progression in the averaged transect from January 2015 to January 2017. Solid line contours are for 100, 200 and 300 meters; the dashed contour indicates 50 meters. Brown and yellow circles represent the winter and spring stations, respectively.

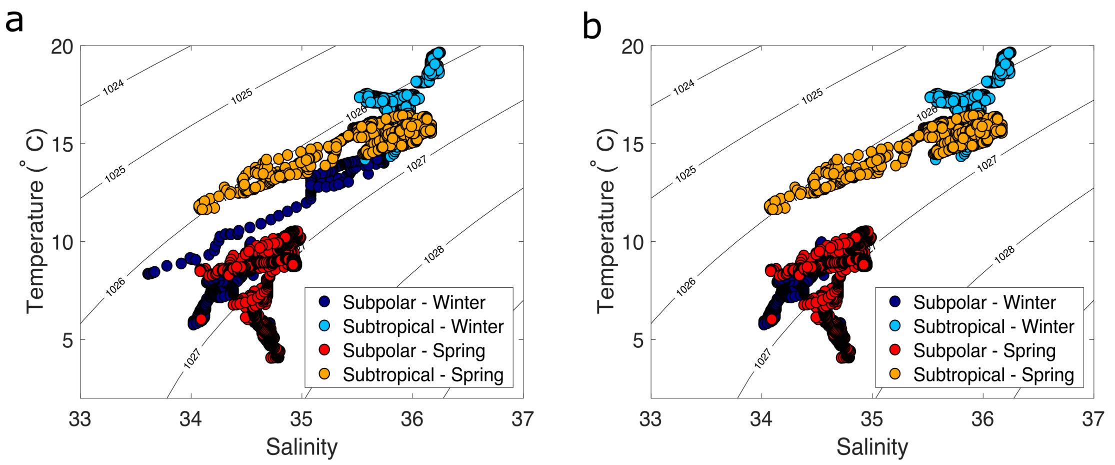

Figure S2 Temperature-Salinity (T-S) diagram of the water masses sampled during the NAAMES1 and NAAMES2 research expeditions. Diagrams were compiled using the near-surface sampling from the onboard thermosalinograph for the times corresponding to the stations described in this manuscript. Blue (Subpolar) and cyan (Subtropical) represent data retrieved in winter (NAAMES1). Red (Subpolar) and yellow in spring (NAAMES2). Black lines represent the contours of constant density. (a) Plot including datapoints from winter station 1 (colored blue by geographical location). (b) Plot excluding datapoints from winter station 1.

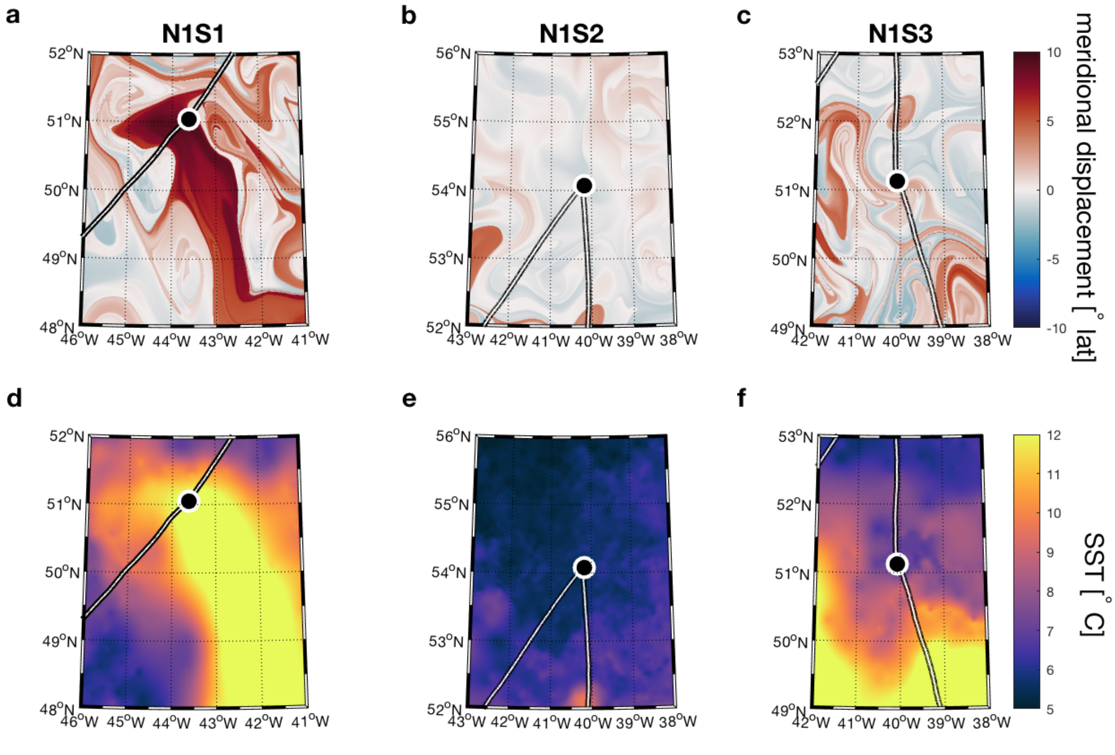

Figure S3 Spatial track of the sampled water mass at the three most northern stations in winter. Station 1 (N1S1), station 2 (N1S2) and station 3 (N1S3). (a,b,c): meridional displacement over 30 days calculated for N1S1 (2015/11/11), N1S2 (2015/11/12) and N1S3 (2015/11/15). The red color identifies water parcels moving from south to north and the blue color vice versa. (d,e,f): Corresponding images of Sea Surface Temperature (SST). Black lines define the trajectory of the R/V Atlantis and black dots the respective stations.

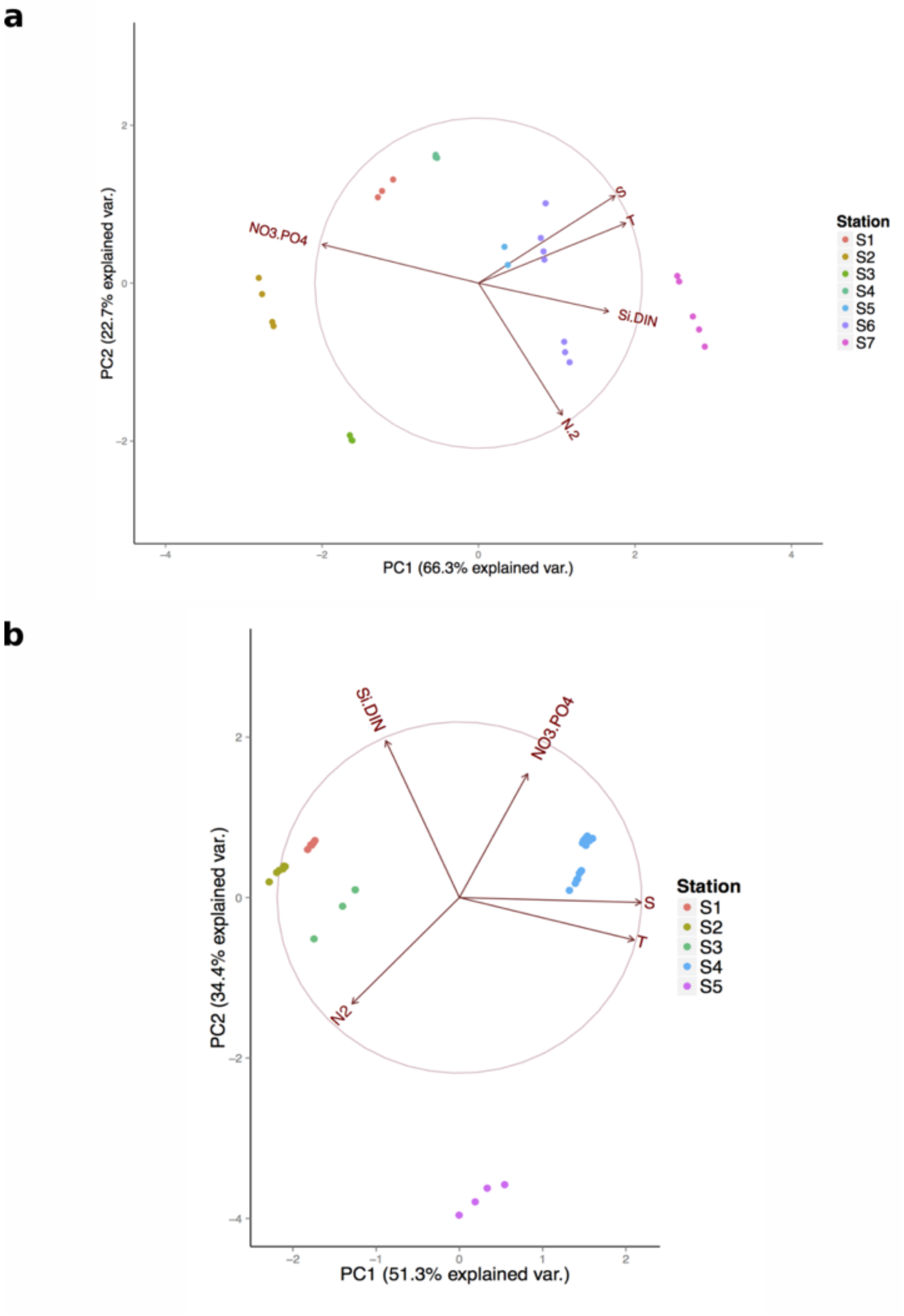

Figure S4 Principal component analysis (PCA) of the station's physical and chemical measurements taken at different depths through NAAMES 1 and 2 stations. T=temperature, S = silicate, N.2 =Nitrogen, Si:DIN= silicate to total dissolved nitrogen, NO3.PO4 = nitrate to phosphate ratio. Samples are color coded by station. (a) Measurements taken in NAAMES 1 (winter) and (b) in NAAMES 2 (spring). Si.DIN is the average 100m ratio of Silicate to total nitrogen and NO3.PO4 is the average 100m ratio of nitrate to phosphate.

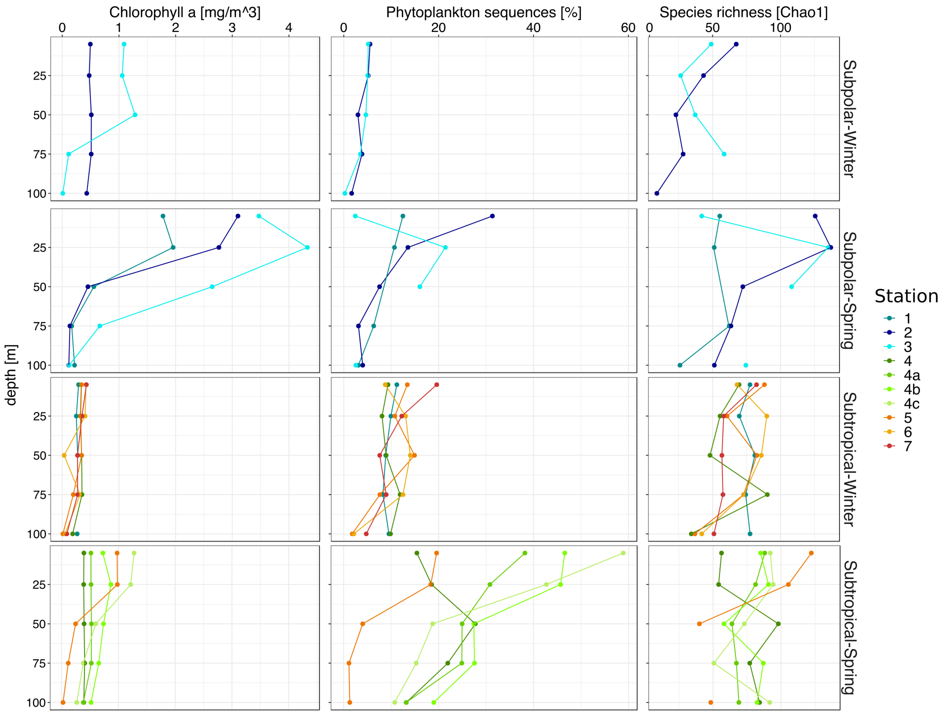

Figure S5 Water column depth profiles of biological properties during NAAMES. Profiles are grouped by categorical classification as the ASVs profile clustering. Chlorophyll a concentration (mg/m3), percentage of retrieved photosynthetic origin sequences from the amplicon dataset, and Chao1 diversity index through the water column estimated from the phytoplankton subset are shown.

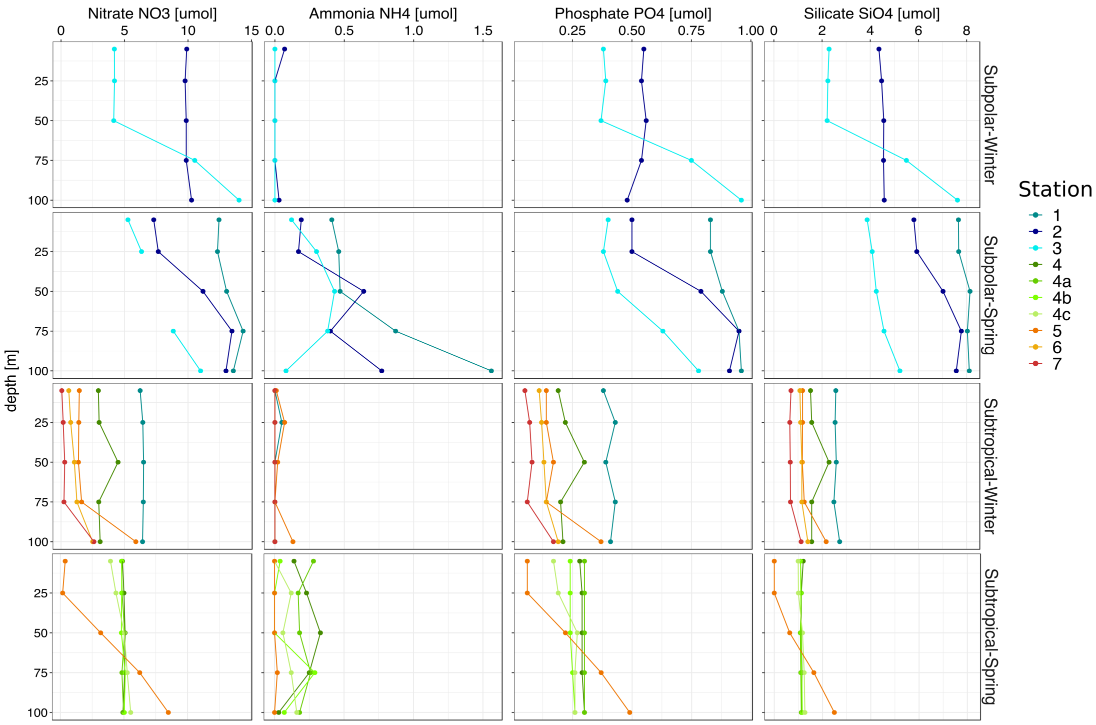

Figure S6 Water column depth profiles of chemical properties during NAAMES. Profiles are grouped by categorical classification as the ASVs profile clustering. Nitrate (NO3), Ammonia (NH4), Phosphate (PO4) and Silicate (SiO4) concentrations.

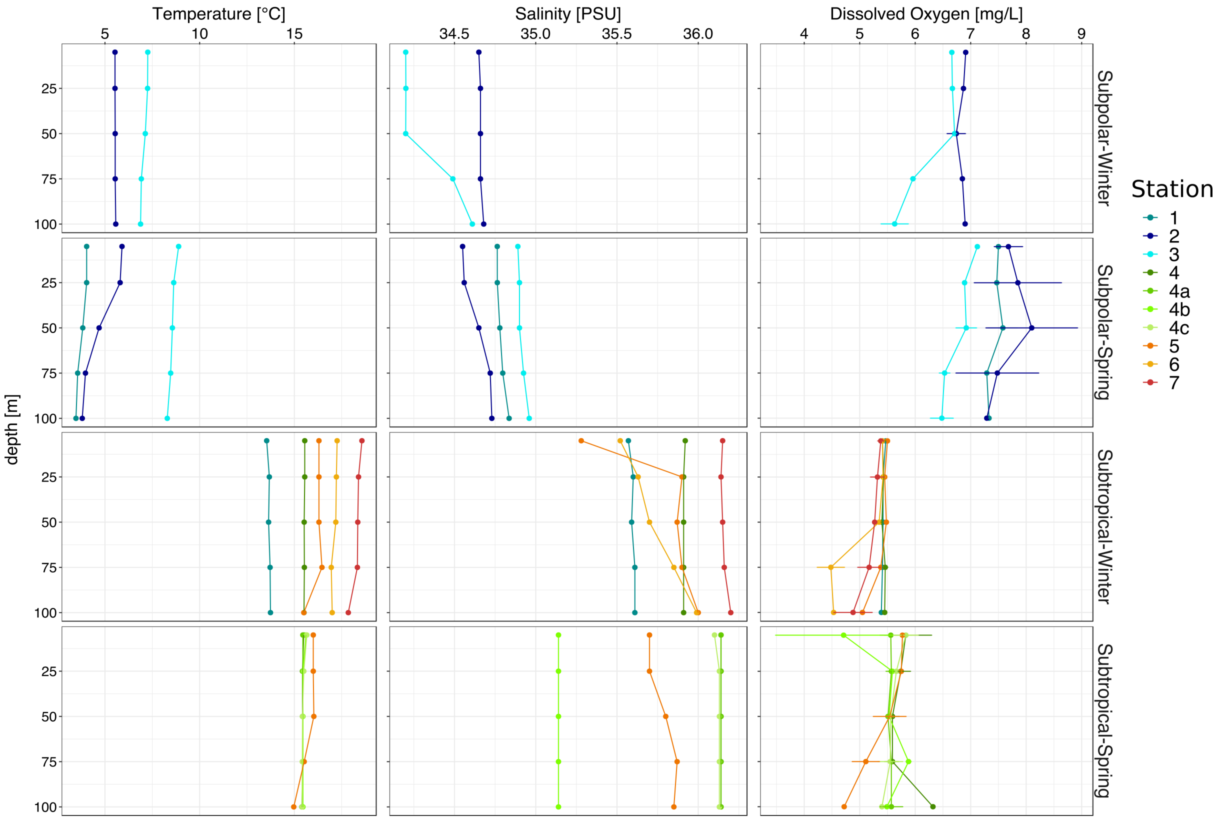

Figure S7 Water column depth profiles of physical properties during NAAMES. Profiles are grouped by categorical classification as the ASVs profile clustering. Temperature, salinity and dissolved oxygen concentrations.

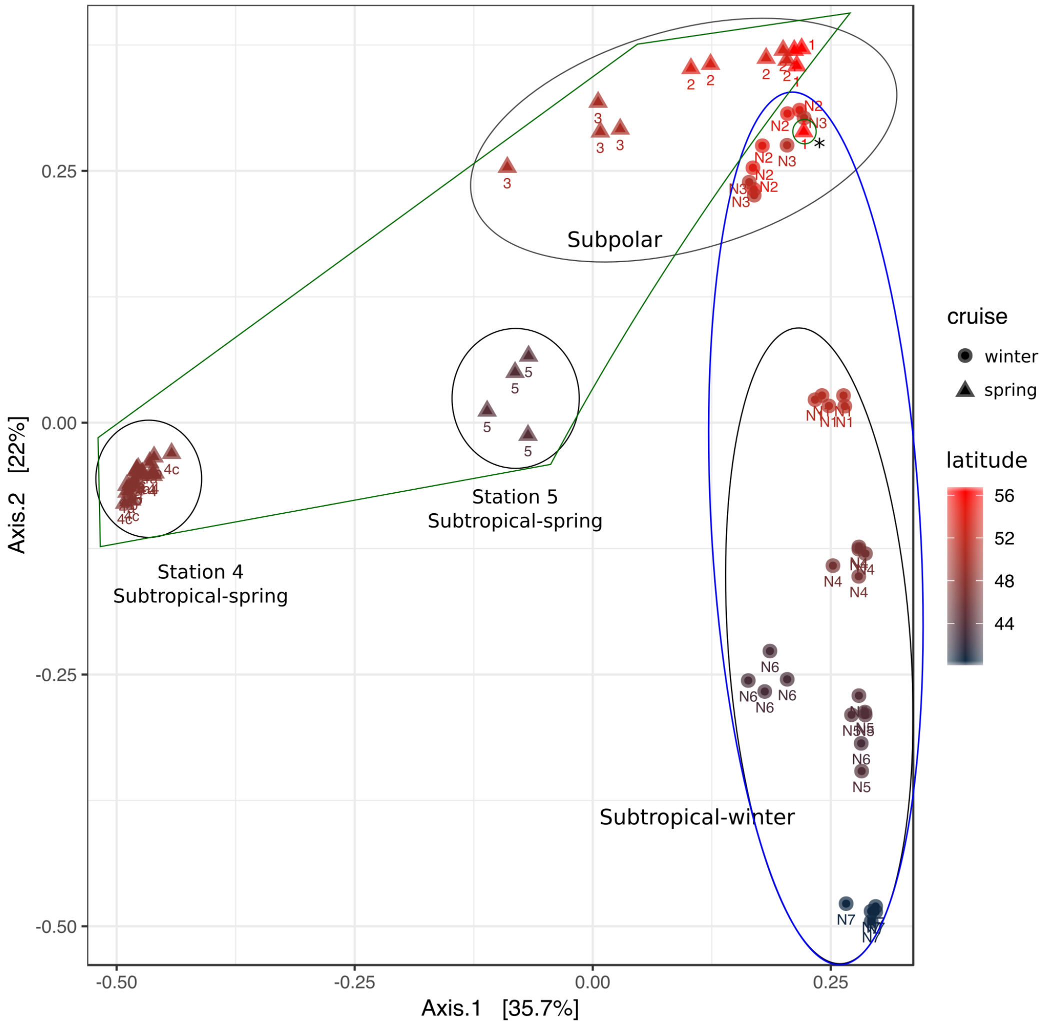

Figure S8 Principal coordinate analysis (PCoA) of the Bray-Curtis dissimilarities between samples. Each dot represents an individual sample and the belonging station is indicated with an adjacent number, the shape of the dot represents the season. Green polygon surrounds winter samples and blue oval the spring samples. The red to blue color gradient represents the north to south latitude in the meridional transects. Asterisk highlights the position of station 1 - 100 m spring sample, which clustered with winter collection in the ASVs hierarchical dendogram.

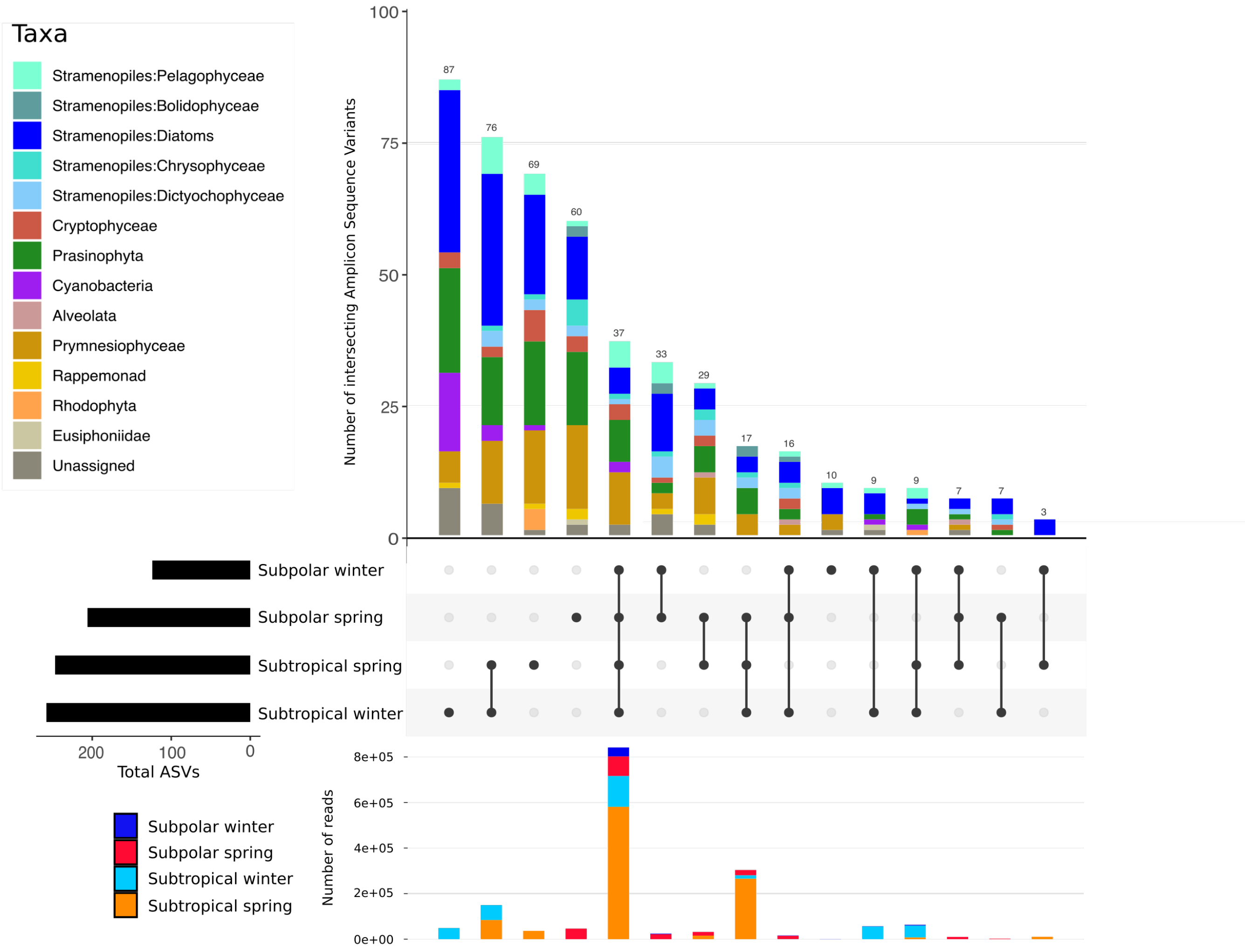

Figure S9 Taxonomic distributions of intersecting ASVs based on the four established categories. "Upset" diagram showing all possible relations between the communities four categories, analogue to a Venn diagram. The top bar plot represents the taxonomic distributions of the ASVs shared in all the possible categorical intersections, unions and relative complements. Intersections, unions and relative complements are shown with black dots in the middle part of the graph. The total number of ASVs found in each category is displayed as horizontal bar plots on the left side of the category name. The bottom bar plot represents the total number of reads for each intersection and is color coded by region-season category. Phytoplankton ASVs common among all seasons represent 39.2% of the total in the subpolar and 40.7% in the subtropical region. Additionally, we identified 37 ASVs (7.67% of the total collection) present in all regions and seasons. This "core" group is taxonomically diverse, indicating that adaptation to variation in environmental conditions leading to high net average success across the system is not a property that is exclusive to broad taxonomic categories. A prominent exception to the generality of broad taxonomic representation across seasons and regions emerged in the strong bias favoring Cyanobacteria in winter in both the subpolar and subtropical. Overall, more than 90% of the total amplicon sequences belonged to intersecting groups that included at least one spring and winter component.

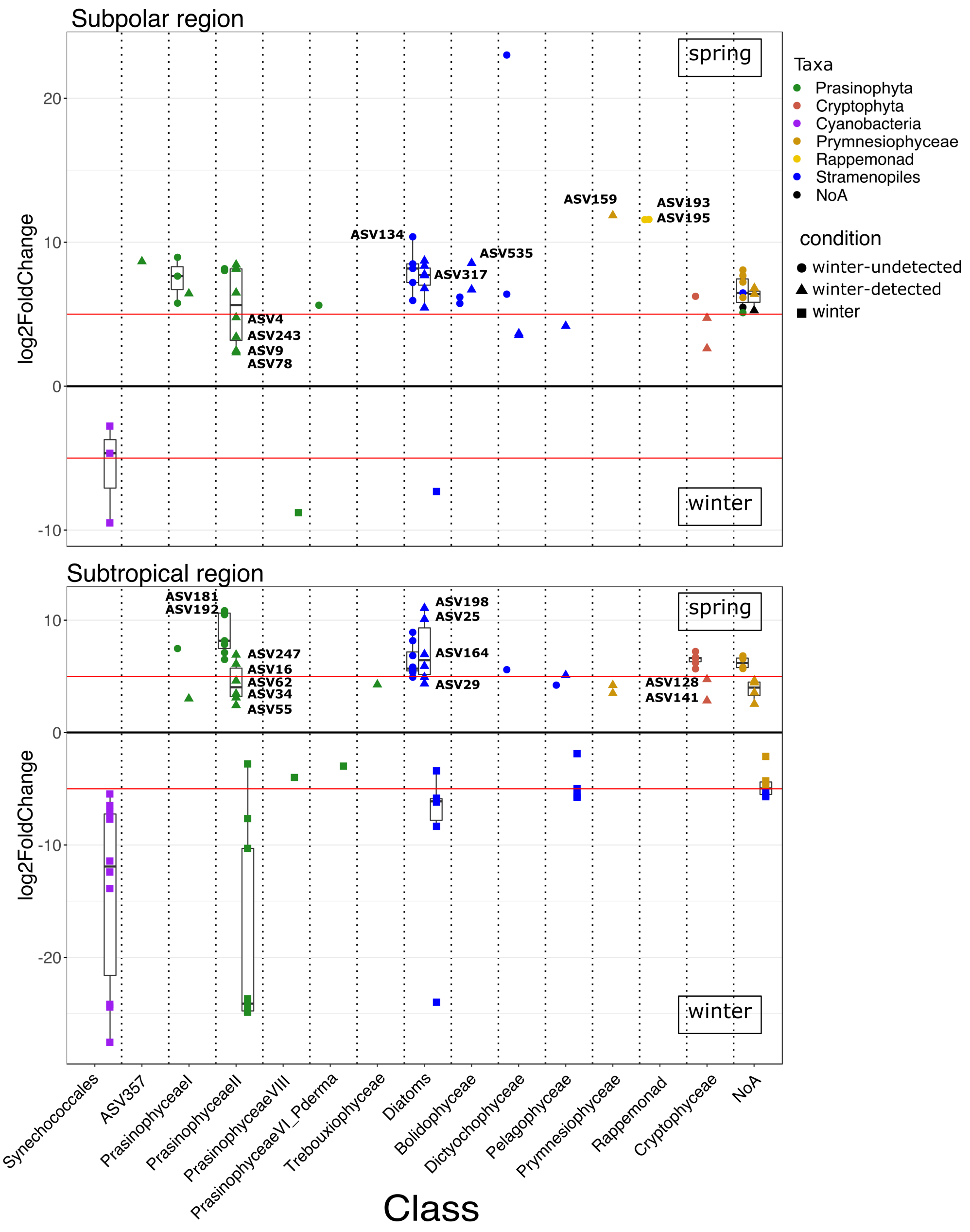

Figure S10 Regional differential abundance of winter-detected and winter-undetected ASVs. Differential abundant ASVs ($p < 0.01$) between winter and spring in the subpolar (top panel) and subtropical (bottom panel) regions. ASVs are organized by taxonomic Class in the x-axis and color coded by major taxonomic grouping. Y-axis indicates the log2 fold change of the differential significant ASVs between the two seasons measured. Red lines are marked at 5 and -5 log2 fold change as a reference. Enriched ASVs in spring are above the black line (positive) and below in winter (negative). Shape of the ASVs shows the condition of being winter-undetected or winter-detected. Elements labeled with their identification number (only for spring-enriched) represent those with a value greater than 100 as base mean (the mean normalized read count for each season), indicating not only a significant differential abundance but a high count of sequences.

TABLE II:

Table S2 Taxonomic assignment of significant (p-value < 0.01) and abundant (baseMean >100) spring enriched (log2fold change > 2) ASVs in both subregions (Fig S10)

Subpolar					
ASV id	baseMean	log2FC	p value	Phylum,Class,Order,Family,Genus,Species	condition
ASV134	123.13	10.38	9.52E-16	Eukaryota,Stramenopiles,Bacillariophyceae,Chaetocerotales,Chaetocerotaceae,Chaetoceros	winter-undetected
ASV195	281.18	11.57	2.58E-13	Eukaryota,Rappemonad,,,,	winter-undetected
ASV193	284.27	11.58	1.85E-10	Eukaryota,Rappemonad,,,,	winter-undetected
ASV4	101.17	4.77	0.000218063	Eukaryota,Chlorophyta,PrasinophyceaeII,Mamiellales,Bathycoccaceae,Ostreococcus,OstreococcusI	winter-detected
ASV535	118.06	10.32	4.91E-21	Eukaryota,Stramenopiles,Bolidophyceae,,,,	winter-detected
ASV128	158.22	2.62	0.000172144	Eukaryota,Cryptophyta,Cryptophyceae,Pyrenomonadales,Geminigeraceae,Teleaulax,	winter-detected
ASV317	174.76	7.69	8.20E-14	Eukaryota,Stramenopiles,Bacillariophyceae,,,,	winter-detected
ASV78	221.28	2.33	0.003578832	Eukaryota,Chlorophyta,PrasinophyceaeII,Mamiellales,Bathycoccaceae,Bathycoccus,	winter-detected
ASV243	337.18	3.42	1.53E-05	Eukaryota,Chlorophyta,PrasinophyceaeII,Mamiellales,Mamiellaceae,Micromonas,MicromonasE2	winter-detected
ASV159	343.68	11.86	6.21E-16	Eukaryota,Haptophyta,Prymnesiophyceae,Phaeocystales,Phaeocystaceae,Phaeocystis,	winter-detected
ASV9	1922.76	2.46	0.000531629	Eukaryota,Chlorophyta,PrasinophyceaeII,Mamiellales,Mamiellaceae,Micromonas,MicromonasABC_C	winter-detected
					winter-detected
Subtropical					
ASV id	baseMean	log2FC	p value	Phylum,Class,Order,Family,Genus,Species	condition
ASV247	127.61	10.49	1.49E-67	Eukaryota,Chlorophyta,PrasinophyceaeII,Mamiellales,Bathycoccaceae,Bathycoccus,	winter-undetected
ASV192	154.84	10.77	4.39E-69	Eukaryota,Chlorophyta,PrasinophyceaeII,Mamiellales,Bathycoccaceae,Ostreococcus,OstreococcusI	winter-undetected
ASV181	162.77	10.84	2.90E-69	Eukaryota,Chlorophyta,PrasinophyceaeII,Mamiellales,Bathycoccaceae,Ostreococcus,OstreococcusI	winter-undetected
ASV128	158.64	2.84	1.61E-09	Eukaryota,Cryptophyta,Cryptophyceae,Pyrenomonadales,Geminigeraceae,Teleaulax	winter-detected
ASV141	191.45	4.74	7.45E-12	Eukaryota,Cryptophyta,Cryptophyceae,Pyrenomonadales,Geminigeraceae,Teleaulax	winter-detected
ASV198	191.29	11.07	1.84E-101	Eukaryota,Stramenopiles,Bacillariophyceae,Chaetocerotales,Chaetocerotaceae,Chaetoceros,	winter-detected
ASV164	225.6	6.96	3.68E-36	Eukaryota,Stramenopiles,Bacillariophyceae,,,,	winter-detected
ASV29	1170.3	4.92	8.45E-64	Stramenopiles,Bacillariophyceae,Cymatosirales,Cymatosiraceae,,	winter-detected
ASV25	1193.79	10.1	1.62E-37	Stramenopiles,Bacillariophyceae,Cymatosirales,Cymatosiraceae,Minutocellus,	winter-detected
ASV62	448.19	3.45	1.66E-18	Eukaryota,Chlorophyta,PrasinophyceaeII,Mamiellales,Bathycoccaceae,Bathycoccus,	winter-detected
ASV55	548.06	2.43	0.000159081	Eukaryota,Chlorophyta,PrasinophyceaeII,Mamiellales,Mamiellaceae,Micromonas,	winter-detected
ASV34	771.15	3.11	3.68E-28	Eukaryota,Chlorophyta,PrasinophyceaeII,Mamiellales,Mamiellaceae,Micromonas,MicromonasE2	winter-detected
ASV16	1274.51	4.61	2.77E-20	Eukaryota,Chlorophyta,PrasinophyceaeII,Mamiellales,Mamiellaceae,Micromonas,	winter-detected
ASV10	1652.16	6.92	3.67E-28	Eukaryota,Chlorophyta,PrasinophyceaeII,Mamiellales,Bathycoccaceae,Ostreococcus,OstreococcusI	winter-detected
ASV4	4454.18	6.1	1.42E-46	Eukaryota,Chlorophyta,PrasinophyceaeII,Mamiellales,Bathycoccaceae,Ostreococcus,OstreococcusI	winter-detected

Phytoplankton size classification and metrics

The most common convention of binning phytoplankton by size defines phytoplankton smaller than 2 μm as 'pico', phytoplankton in the size range 2-20 μm as 'nano', and phytoplankton in the size range 20-200 μm as 'micro' (Sieburth and Smetaceck 1978). It is based on the use of one dimension as a size descriptor, typically the equivalent spherical diameter (ESD) or major axis length (MLA). ESD is commonly used when size is not directly measured (e.g., coulter counter, flow-cytometers, optical measurements) and theoretical or empirical models that assume spherical particles are used to derive size from a physical measurement (e.g., electrical impedance or light scattering). Other metrics of size such as a pervalvar and/or apical axis length have been used for organisms with elongated shape (e.g. Thalassionema, Pseudo-nitzschia), and for organisms with more complex shapes (e.g. Ceratiums, Gymnodinium, Phalacroma) a scale bar is placed on the side of the drawing but no length is reported (Hasle et al. 1997). The choice of size metrics strongly affects the size classification of phytoplankton especially for populations of phytoplankton that diverge from spherical shapes and may bias the ecological interpretation of field observations.

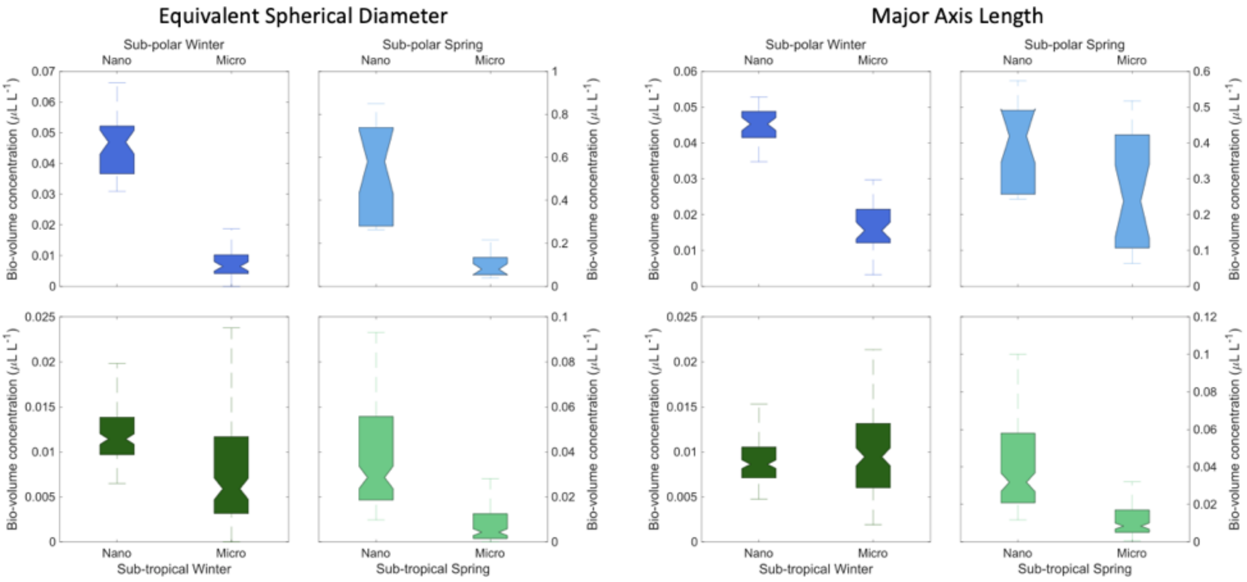

Figure S11 Comparison of the effect of two size metrics. Equivalent spherical diameter (left) and major axis length (right) contributions of nano- and micro-phytoplankton to the total bio-volume.

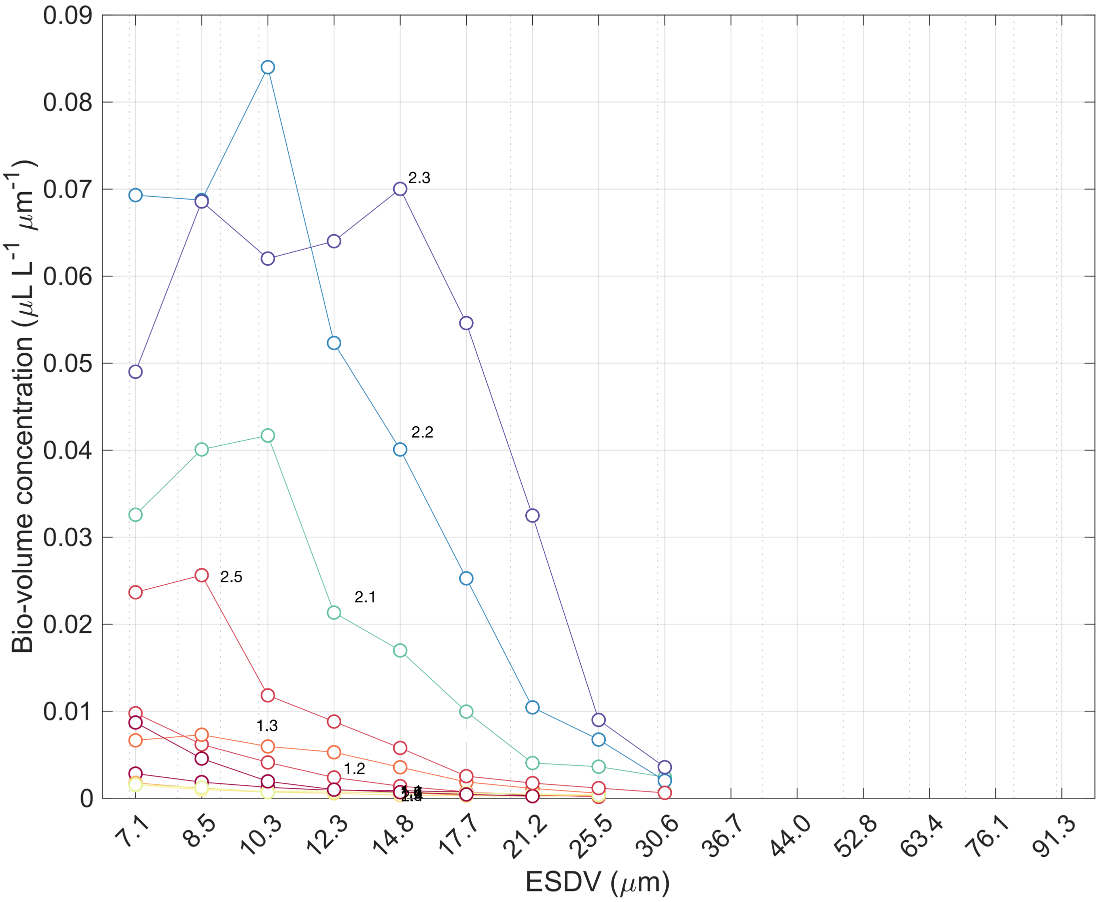

Figure S12 Total bio-volumes distributions at the surface (ship intake) by cell-size were derived from the IFCB (fraction of cells >8 μm diameter) at each station for both campaigns. Individual measurements for each station surface water is plotted as cell bio-volume per unit volume of seawater (bio-volume concentration). Individual station distributions are shown in different colors and labeled over each curve. Average of these distributions by region and season is shown in the main text (Figure 3b).
